# Advances in Clinical Voice Quality Analysis with VOXplot

**DOI:** 10.3390/jcm12144644

**Published:** 2023-07-12

**Authors:** Ben Barsties v. Latoszek, Jörg Mayer, Christopher R. Watts, Bernhard Lehnert

**Affiliations:** 1 Speech-Language Pathology, SRH University of Applied Health Sciences, 40210 Düsseldorf, Germany; 2 Institute for Natural Language Processing, University of Stuttgart, 70049 Stuttgart, Germany; jmayer@lingphon.net; 3 Harris College of Nursing & Health Sciences, Texas Christian University, Fort Worth, TX 76109, USA; 4 Department of Oto-Rhino-Laryngology, Phoniatrics and Pedaudiology Division, University Medicine Greifswald, 17475 Greifswald, Germany

**Keywords:** voice quality analysis, voice diagnostic, acoustic measures, hoarseness, breathiness

## Abstract

Background: The assessment of voice quality can be evaluated perceptually with standard clinical practice, also including acoustic evaluation of digital voice recordings to validate and further interpret perceptual judgments. The goal of the present study was to determine the strongest acoustic voice quality parameters for perceived hoarseness and breathiness when analyzing the sustained vowel [a:] using a new clinical acoustic tool, the VOXplot software. Methods: A total of 218 voice samples of individuals with and without voice disorders were applied to perceptual and acoustic analyses. Overall, 13 single acoustic parameters were included to determine validity aspects in relation to perceptions of hoarseness and breathiness. Results: Four single acoustic measures could be clearly associated with perceptions of hoarseness or breathiness. For hoarseness, the harmonics-to-noise ratio (HNR) and pitch perturbation quotient with a smoothing factor of five periods (PPQ5), and, for breathiness, the smoothed cepstral peak prominence (CPPS) and the glottal-to-noise excitation ratio (GNE) were shown to be highly valid, with a significant difference being demonstrated for each of the other perceptual voice quality aspects. Conclusions: Two acoustic measures, the HNR and the PPQ5, were both strongly associated with perceptions of hoarseness and were able to discriminate hoarseness from breathiness with good confidence. Two other acoustic measures, the CPPS and the GNE, were both strongly associated with perceptions of breathiness and were able to discriminate breathiness from hoarseness with good confidence.

## 1. Introduction

Standard clinical practice for the evaluation of voice disorders includes a battery of multidimensional assessments (e.g., visual analysis, auditory-perceptual judgment, aerodynamic analysis, acoustic analysis, and self-assessment [1]) aimed to describe and diagnose the voice complaint. Voice disorders affect quality, volume, pitch, resonance, flexibility, and/or stamina. These vocal changes are the manifestation of disordered respiratory, laryngeal, and vocal tract functions, which might result, in many cases, from heterogeneous local etiologies [2]. Many voice disorders are associated with abnormal oscillation patterns of the vocal folds. The resulting voiced energy can vary as a function of vibrational changes at different vocal fold areas, but especially at the free vocal fold margin. Furthermore, the more a critical region of one vocal fold or both vocal folds are affected by laryngeal pathology, the more variation in vocal sound energy and subsequent perceptions of voice quality severity can be expected [3].

Although voice quality is not a clearly defined term, there are two general approaches to evaluation [4]. First, the subjective approach of listening to the patient’s voice and assigning a score to different perceptual domains is considered a gold standard approach for perceptual voice analysis. Second, the use of an objective instrumental approach can be used, in which a specific computer algorithm is applied to recorded voice signals. Examples of instrumental assessment of voice quality include analysis of the acoustic voice sound signal and the inverse-filtered oral airflow signal or its derivative. Although many different terms have been used to describe voice quality, a wide acceptance has been acknowledged for terms such as hoarseness or overall voice quality, and major subtypes of the general anomalies in voice quality such as breathiness, roughness, and strain [4,5].

An objective acoustic analysis of voice signals is the most commonly used instrumental tool in clinical practice and research for objectively characterizing voice disorders [6]. Voice signals can be analyzed acoustically in the domains of time, frequency, amplitude, and quefrency. A large number of acoustic measures have been introduced and described to objectively predict dysphonia types and severities. This is illustrated in a taxonomy by Buder [6] with 15 signal-processing-based categories. The reliable and valid use of objective acoustic analysis in research or clinical practice depends on specific requirements (e.g., hardware, software, and examination circumstances) to enable voice analysis with high accuracy and reliability [4,7].

The quantification of voice quality with acoustic methods has traditionally been analyzed on sustained vowels. Although the assessment of voice quality based on sustained vowels (SV) does not necessarily correspond to that of continuous speech (CS) [8,9], acoustic measures from sustained vowels are ubiquitous in research and clinical practice. Acoustic parameters that correlate strongly with auditory-perceptual judgments are included in two examples of multiparametric acoustic indices: the acoustic voice quality index (AVQI) for the evaluation of hoarseness, and the acoustic breathiness index (ABI), which assesses the hoarseness subtype, breathiness [10]. Both AVQI and ABI have been used with wide international acceptance for research and clinical practice for a number of reasons: (a) their multivariate constructs based on linear regression analysis that combines relevant acoustic markers; (b) the inclusion of both continuous speech and sustained vowels in the acoustic analysis; (c), signal processing that uses algorithms of the freeware Praat; and (d) a single score ranging from 0 to 10 for the entire recording being analyzed (i.e., the higher AVQI or ABI score, the more severe the related anomaly of voice quality, and vice versa) [10].

The acoustic measures of AVQI and ABI include smoothed cepstral peak prominence (CPPS); harmonics-to-noise ratio (HNR); shimmer percentage; shimmer dB; general slope of the spectrum (Slope); and tilt of the regression line through the spectrum (Tilt); jitter local; glottal-to-noise excitation ratio with a maximum frequency of 4500 Hz (GNE); relative level of high-frequency noise between energy from 0 to 6 kHz and energy from 6 to 10 kHz (HF Noise); HNR by Dejonckere (HNR-D), which analyses the harmonic shape of the spectral display by using the frequency bandwidth between 500 and 1500Hz and a cepstrum to determine F0, and thus locate the harmonic structure in the long-term average of the spectrum; differences between the amplitude of the first and second harmonics in the spectrum (H1H2); and period standard deviation(PSD).

Next to AVQI and ABI, a third multivariate index with a long tradition in the evaluation of overall voice quality on sustained vowels is the dysphonia severity index (DSI) [11,12]. The DSI includes four voice parameters (jitter local; highest frequency and lowest intensity of a voice range profile; and maximum phonation time), in which jitter local is the only acoustic single parameter directly associated with voice quality. To use the DSI with Praat algorithms for signal processing the pitch perturbation quotient was considered in place of jitter local [13].

VOXplot (Lingphon, Straubenhardt, Germany; https://voxplot.lingphon.com, accessed on 11 June 2023) is a new freeware application for acoustic voice quality analysis based on the Praat algorithms for signal processing. Whereas Praat is a versatile and correspondingly complex software for acoustic analysis of arbitrary signals, VOXplot is specifically tailored to the analysis of voice quality. With Praat, only the algorithms are used, while the user interface of VOXplot is designed to meet the demands of standardized and intuitive ease of use for clinicians and researchers. VOXplot covers the entire workflow of acoustic voice quality assessment: recording and recording quality assessment, acoustic voice quality analysis, and generation of a concise PDF (or JPEG/PNG) sheet with the analysis results. The core analysis of VOXplot is the voice quality analyses of continuous speech and sustained vowels with AVQI and ABI. VOXplot is currently available in 12 analysis languages for AVQI and ABI, which are based on more than one decade of research knowledge [14,15]. The validation results of both indices relate only to an objective evaluation of the hoarseness and breathiness levels for heterogeneous voice disorders in comparison with vocally healthy volunteers with no further specification of a specific disorder or vocal symptom. The usability of VOXplot is currently available in three interface languages. Further details of sustained vowels can be analyzed qualitatively with the narrowband spectrogram and quantitatively with single acoustic parameters.

As mentioned before, AVQI, ABI, and DSI are used in combination with highly sensitive acoustic markers for the evaluation of hoarseness and breathiness. However, a direct comparison of these objective metrics using the VOXplot application with perceptual ratings of hoarseness or breathiness is missing. Therefore, the aim of this study was to compare the concurrent validity and diagnostic validity outcomes of 13 single acoustic voice quality measures between hoarseness and breathiness aspects on sustained vowels.

## 2. Materials and Methods

### 2.1. Participants

In the present study, the voice recordings and auditory-perceptual judgment of hoarseness and breathiness acquired in a previous study [16] were applied to new analyses. The group of dysphonic participants consisted of 175 patients with various organic and nonorganic voice disorders and various degrees of dysphonia severity. The control group of 43 vocally healthy volunteers reported no voice complaints, history of voice, speech, or hearing problems, and no impact of voice problems as measured with the voice handicap index [17].

Table 1 summarizes the demographic data and the types of dysphonia for the two groups. For further details regarding the data and recording acquisition, and inclusion and exclusion criteria, we refer to Barsties v. Latoszek et al. (2020) [16].

All the participants gave their informed consent for inclusion before they participated in the study. The study was conducted in accordance with the Declaration of Helsinki, and the protocol was approved by the Ethics Committee of Greifswald University (BB072/16).

### 2.2. Auditory-Perceptual Judgment

For the auditory-perceptual judgment ratings, a panel of three male experts specialized in voice disorders with experience ranging from 8 to 31 years was used. The GRBAS scale was used for data collection. Each listener rated ordinally on a four-point scale the hoarseness level, which is represented in the G-parameter (Grade), and the breathiness severity, which is represented in the B-parameter (which represents the degree of the extent of air leakage through the glottis).

For further details regarding the rating scale, rating procedure, anchor voices, reliability results of the raters, and deviation of the rating level results from the expert panel for hoarseness and breathiness, we refer to Barsties v. Latoszek et al. (2020) [16].

### 2.3. Acoustic Measurements

The acoustic analyses were conducted only on recordings of the sustained vowel [a:] across 3 s of the mid-vowel segment from a single trial. The [a:] vowel was used as a typical open front vowel for the clinical and scientific acoustic tasks, which is easily recognized regardless of the native language, linguistic competence, or individual health problems (e.g., hearing disorders) from the test person in comparison to other vowels [18,19]. These sound files were applied to a new analysis using VOXplot. In total, 13 single voice quality parameters were acquired from each recording, which are listed in Table 2.

### 2.4. Statistics

The association of the 13 acoustic parameters with the two auditory-perceptual evaluations of hoarseness and breathiness from 218 recorded voice samples was investigated by calculating Spearman’s rank correlation coefficients. An absolute correlation score of ≥0.70 is marked as a high relationship for the concurrent validity aspect between the acoustic parameter and the perceived voice quality evaluation [20].

The Fisher r-to-z transformation was used to assess the statistical significance of the two correlation coefficients from the outcomes of the acoustic parameter and perceived hoarseness vs. perceived breathiness levels.

A receiver operating characteristic (ROC) curve was then generated in order to analyze the diagnostic accuracy of the 13 acoustic metrics according to sensitivity (results of the participants with hoarseness or breathiness) and specificity (results of participants without hoarseness or breathiness). The power of the acoustic markers to discriminate between the absence and presence of hoarseness or breathiness was estimated using the area under the ROC curve (A_ROC_). An A_ROC_ of >0.90 is considered to be exceptionally good; an A_ROC_ of <0.70 is considered to be low, and an A_ROC_ of ≤ 0.50 corresponds to a chance level of diagnostic accuracy [21]. In order to find the optimal threshold value that best differentiates between without and with hoarseness or breathiness, the Youden index (a measure that uses a receiver operating characteristic to determine which threshold value is best suited to distinguish two groups in a measurement) was calculated as sensitivity + specificity − 1.

The significant differences between the two ROC curves (calculated for hoarseness and breathiness) of the acoustic measures were determined by the difference between the areas under the curves [22].

The statistical analyses were performed using SPSS, version 23, for Windows (IBM Corp., Armonk, NY, USA). The tests of significance between the two correlation coefficients and between the areas under two independent ROC curves were analyzed on VassarStats (R. Lowry, Vassar College, NY, USA, 1998–2023; http://vassarstats.net/, accessed on 11 June 2023). Results were considered statistically significant at *p* ≤ 0.05.

## 3. Results

Table 3 presents the validation outcomes for the 13 single acoustic voice quality parameters in direct comparison to the auditory-perceptual ratings of hoarseness and breathiness. The thresholds with sensitivity and specificity, based on the ROC statistics and the Youden Index, are also listed in Table 3.

For hoarseness, a strong correlation was present for CPPS, HNR, and PPQ5. No acoustic parameter reached an exceptionally good level of A_ROC_, and 4 of the 13 acoustic parameters revealed a low level of A_ROC_, in which one of them was characterized by a chance level in diagnostic accuracy (H1H2).

For breathiness, a strong correlation was present for CPPS and GNE. However, GNE reached an exceptionally good A_ROC_ result, and 9 of the remaining 12 acoustic parameters had a strong level of diagnostic accuracy.

To assign a single acoustic voice quality parameter with high validity to a type of voice abnormality, (a) the absolute correlation value and the A_ROC_ had to be >0.70, and (b) significant differences in validity performances between hoarseness and breathiness must be obtained in the correlation results or the A_ROC_ outcomes. According to the results listed in Table 3 for hoarseness, two acoustic parameters could be identified as highly valid (HNR and PPQ5) in comparison to breathiness. For breathiness, two acoustic metrics (CPPS and GNE) were also revealed to have outstanding validity results in comparison to hoarseness.

## 4. Discussion

The aim of the present study was to investigate the validity of single acoustic parameters representing voice quality characteristics of hoarseness or breathiness in a direct comparison of the auditory-perceptual voice quality ratings of those domains from sustained vowel [a:] phonation. Although multiparametric models are preferred in highly valid evaluations of hoarseness or breathiness [4,9,23,24], single acoustic parameters are mostly used in clinical practice and recommended protocols for instrumental assessment of voice [7]. The present study attempted to reveal the most relevant acoustic markers for hoarseness and breathiness from a pool of metrics, which are already part of relevant multiparametric models in the evaluation of voice quality, such as DSI, AVQI, and ABI.

In general, the results from the initial AVQI and ABI studies were confirmed by the present study, with comparable results to the correlation coefficients for hoarseness and breathiness [9,24]. Although continuous speech was also considered in the voice quality evaluation for AVQI and ABI, CPPS and HNR showed high agreement for hoarseness, and CPPS and GNE presented the strongest results for breathiness. Because perceptions of breathiness are associated with high irregularity in the acoustic spectrum (e.g., a lot of spectral aperiodicity or noise), while perceptions of hoarseness can be associated with multidimensional acoustic factors other than spectral aperiodicity, it was logical that the discriminative ability of CPPS (which measures the periodicity in the acoustic spectrum) for breathiness was significantly higher than for hoarseness in this study. Originally, CPPS was developed for the vocal quality abnormality of breathiness [25], in which breathiness is a main subtype of hoarseness [24]. Just like GNE, which was also developed for the evaluation of breathiness [26], the present study confirmed its strength in the evaluation of this voice quality aspect with significantly higher concurrent validity and diagnostic accuracy.

A clearer unique identifier for hoarseness versus breathiness was shown in this study by the two parameters HNR and PPQ5. In the case of HNR, it is the second most important acoustic parameter in the AVQI formula after CPPS, which is supported by the results of this study [9]. The findings of this study suggest that HNR is a general parameter that does not necessarily correspond to other strong breathiness measures such as CPPS or GNE. Only PPQ5 achieved a sufficiently high agreement with hoarseness and was significantly differentiated from breathiness in the current study. This result was contrary to the results of the original study on AVQI by Maryn et al. (2010) [9]. Furthermore, in a meta-analysis on the evaluation of hoarseness, jitter parameters generally ranked significantly lower than spectral or cepstral parameters and some shimmer markers [27], but, according to the present results, PPQ5 seems to be robust enough to assess hoarseness in the evaluation of sustained vowels, which may explain why this parameter is included in the DSI formula.

**Figure 1:**
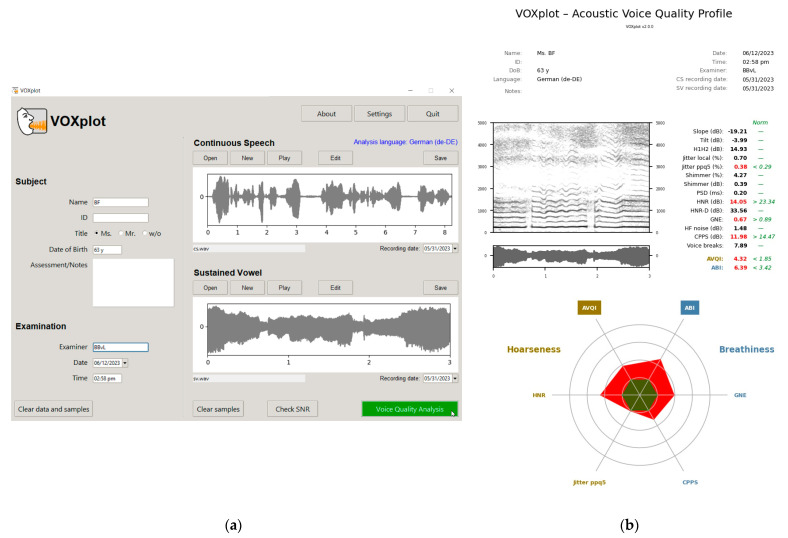
VOXplot version 2.0: (**a**) the user interface for preparing the acoustic analysis of continuous speech and/or sustained vowels selected in the English language with the analysis language German for the thresholds evaluations of AVQI and ABI; (**b**) the outcome of the main voice quality parameters in VOXplot, which are evaluated quantitatively and/or qualitatively for hoarseness and breathiness.

The new developments based on the present study were updated in VOXplot and are available from version 2.0 (see Figure 1).

## 5. Conclusions

For the voice quality evaluation on the sustained vowel HNR and PPQ5 (for hoarseness), and CPPS and GNE (for breathiness) yielded the highest significant validity results compared to each of the other voice quality aspect.” These four acoustic parameters should have priority in the evaluation of hoarseness and breathiness and are prominently included in VOXplot (e.g., in the voice quality circle plot).

## Figures and Tables

**Table 1:** Demographic data and types of voice disorders of the dysphonia and control groups.

Group	Type of Dysphonia	Number	Gender		Age in Years	
			Female	Male	Mean	SD
Dysphonia Group	Carcinoma of head and neck	55	13	42	61.25	10.18
	Functional dysphonia	38	26	12	52.11	16.48
	Larynx carcinoma	28	1	27	69.96	9.05
	Paralyses	25	14	11	63.36	16.09
	Nodules	8	5	3	33.25	19.43
	Reflux laryngitis	4	4	0	54.50	5.45
	Cancer of unknown primary syndrome	4	2	2	61.00	8.21
	Mutational falsetto	3	0	3	15.67	3.06
	Leukoplakia	2	0	2	57.00	8.49
	Granuloma	2	0	2	42.00	11.31
	Laryngitis	2	1	1	39.50	12.02
	Parkinson’s	2	0	2	74.00	11.31
	Polyp	1	0	1	60.00	-
	Laryngeal trauma	1	0	1	78.00	-
Control group	None	43	23	20	26.79	7.06

Abbreviation. SD = standard deviation.

**Table 2:** List of 13 acoustic measures for the voice quality evaluation.

Category	Acoustic Measures	Abbreviation
Fourier and linear prediction coefficient spectra	Smoothed cepstral peak prominence is the distance between the first harmonic peak and the point with equal quefrency on the regression line through the smoothed cepstrum.	CPPS (dB)
	Differences between the amplitudes of the first and second harmonics in the spectrum. To localize the first harmonic peak, a cepstrum was performed for F0 determination.	H1H2 (dB)
	Relative level of high-frequency noise between energy from 0 to 6 kHz and energy from 6 to 10 kHz.	HF-Noise (dB)
	Harmonics-to-noise ratio is the base 10 logarithm of the ratio between the periodic energy and the noise energy, multiplied by 10 HNR.	HNR (dB)
	Harmonics-to-noise ratio from Dejonckere and Lebacq, which analyzes the harmonic emergence of the spectral display comprised within the frequency bandwidth between 500 Hz and 1500 Hz. A cepstrum was performed to determine F0 and thus to localize the harmonic structure in the long-term average spectrum.	HNR-D (dB)
	General slope of the spectrum is defined as the difference between the energy within 0–1000 Hz and the energy within 1000–10,000 Hz of the long-term average spectrum.	Slope (dB)
	Tilt of the regression line through the spectrum is the difference between the energy within 0–1000 Hz and the energy within 1000–10,000 Hz of the trendline through the long-term average spectrum.	Tilt (dB)
Frequency of short-term perturbation measures	Period standard deviation is the variation in the standard deviation of periods in which the length of the sample is important for a valid computation of the standard deviation.	PSD (ms)
Frequency of short-term perturbation measures	Two jitter variations: Jitter local is the average difference between successive periods, divided by the average period.	Jitter local (%)
	Jitter of the five-point period perturbation quotient is the average absolute difference between a period and the average of it and its four closest neighbors, divided by the average period.	PPQ5 (%)
Amplitude of short-term perturbations measures	Two shimmer variations: Shimmer local is the absolute mean difference between the amplitudes of successive periods, divided by the average amplitude.	Shimmer (%)
	Shimmer local dB is the base 10 logarithm of the difference between the amplitudes of successive periods, multiplied by 20.	Shimmer (dB)
Combines spectral and perturbation features	The glottal-to-noise-excitation (GNE) ratio with a maximum frequency of 4500 Hz.	GNE

**Table 3:** Validation results of the 13 single acoustic voice quality parameters of the sustained vowel phonation [a:].

Voice Quality Parameters	Validation Parameters	Hoarseness	Breathiness
CPPS (dB)	Correlation	−0.76 *	−0.81 *
	A_ROC_	0.823 *	0.915 **
	Threshold	15.02 dB	14.47 dB
	Sensitivity	84.7%	88.1%
	Specificity	71.2%	81.7%
GNE	Correlation	−0.70	−0.78 *
	A_ROC_	0.798 *	0.886 *
	Threshold	0.91	0.89
	Sensitivity	88.9%	91.7%
	Specificity	62.3%	74.3%
H1H2 (dB)	Correlation	0.03	0.12
	A_ROC_	0.448	0.584
	Threshold	Chance−level based on A_ROC_	6.39 dB
	Sensitivity	Chance−level based on A_ROC_	40.4%
	Specificity	Chance−level based on A_ROC_	82.6%
HNR (dB)	Correlation	−0.71 *	−0.56
	A_ROC_	0.812 *	0.794 *
	Threshold	23.34 dB	23.34 dB
	Sensitivity	90.3%	78.9%
	Specificity	62.9%	68.5%
HNR-D (dB)	Correlation	−0.57	−0.38
	A_ROC_	0.760 *	0.701 *
	Threshold	31.77 dB	24.23 dB
	Sensitivity	61.1%	77.1%
	Specificity	80.8%	53.2%
HF noise (dB)	Correlation	−0.48	−0.49
	A_ROC_	0.698	0.728 *
	Threshold	2.28 dB	2.29 dB
	Sensitivity	80.6%	77.1%
	Specificity	54.1%	62.4%
Jitter local (%)	Correlation	0.68	0.57
	A_ROC_	0.839 *	0.808 *
	Threshold	0.50%	0.57%
	Sensitivity	70.8%	71.0%
	Specificity	84.7%	78.0%
PPQ5 (%)	Correlation	0.71 *	0.55
	A_ROC_	0.833 *	0.799 *
	Threshold	0.29%	0.32%
	Sensitivity	67.2%	67.0%
	Specificity	84.5%	75.9%
PSD (ms)	Correlation	0.59	0.41
	A_ROC_	0.802 *	0.730 *
	Threshold	0.00012 ms	0.00018 ms
	Sensitivity	65.3%	50.5%
	Specificity	81.9%	88.1%
Shimmer (%)	Correlation	0.65	0.53
	A_ROC_	0.773 *	0.780 *
	Threshold	3.08%	3.58
	Sensitivity	53.5%	57.0%
	Specificity	91.7%	90.8%
Shimmer (dB)	Correlation	0.66	0.55
	A_ROC_	0.783 *	0.786 *
	Threshold	0.27 dB	0.33 dB
	Sensitivity	54.9%	57.9%
	Specificity	91.7%	91.7%
Slope (dB)	Correlation	−0.09	−0.11
	A_ROC_	0.617	0.602
	Threshold	−25.08 dB	−25.34 dB
	Sensitivity	81.9%	80.7%
	Specificity	39.7%	43.1%
Tilt (dB)	Correlation	0.30	0.43
	A_ROC_	0.592	0.673
	Threshold	−10.32 dB	−11.73 dB
	Sensitivity	34.9%	81.7%
	Specificity	86.1%	46.8%

* High correlation or high A_ROC_ indicating a marked relationship in concurrent validity or sufficient diagnostic accuracy; ** exceptionally good diagnostic accuracy level. Darker grey boxes indicate nonsignificant differences of *p* > 0.05 (corresponding to Fisher r-to-z transformation for correlation results and/or significant differences in ROC results of A_ROC_).

## Data Availability

The original contributions presented in the study are included in the article; further inquiries can be directed to the corresponding author.
